# Strategies to promote treatment compliance: a grounded theory study with relatives of people with a serious mental health condition

**DOI:** 10.1186/s12888-024-05907-9

**Published:** 2024-07-08

**Authors:** Christin Hempeler, Sarah Potthoff, Matthé Scholten, Georg Juckel, Jakov Gather

**Affiliations:** 1 Institute for Medical Ethics and History of Medicine, Ruhr University Bochum, Markstraße 258a, 44799 Bochum, Germany; 2 Department of Psychiatry, Psychotherapy and Preventive Medicine, LWL University Hospital, Ruhr University Bochum, Alexandrinenstraße 1-3, 44791 Bochum, Germany; 3 Institute for Experimental Medicine, Department for Medical Ethics, Christian-Albrechts University Kiel, Preusserstraße 1-9, 24105 Kiel, Germany

**Keywords:** Treatment pressure, Psychological pressure, Informal coercion, Informal caregiving, Informed consent, Voluntariness, Ethics

## Abstract

**Background:**

Treatment pressures encompass communicative strategies that influence mental healthcare service users’ decision-making to increase their compliance with recommended treatment. Persuasion, interpersonal leverage, inducements, and threats have been described as examples of treatment pressures. Research indicates that treatment pressures are exerted not only by mental healthcare professionals but also by relatives. While relatives play a crucial role in their family member’s pathway to care, research on the use of treatment pressures by relatives is still scarce. Likewise, little is known about other strategies relatives may use to promote the treatment compliance of their family member with a serious mental health condition. In particular, no study to date has investigated this from the perspective of relatives of people with a serious mental health condition.

**Aim:**

The aim of this study was to answer the following research questions: Which types of treatment pressures do relatives use? Which other strategies do relatives use to promote the treatment compliance of their family member with a serious mental health condition? How do treatment pressures relate to these other strategies?

**Methods:**

Eleven semi-structured interviews were conducted with relatives of people with a serious mental health condition in Germany. Participants were approached via relatives’ self-help groups and flyers in a local psychiatric hospital. Inclusion criteria were having a family member with a psychiatric diagnosis and the family member having experienced formal coercion. The data were analyzed using grounded theory methodology.

**Results:**

Relatives use a variety of strategies to promote the treatment compliance of their family member with a serious mental health condition. These strategies can be categorized into three general approaches: influencing the decision-making of the family member; not leaving the family member with a choice; and changing the social or legal context of the decision-making process. Our results show that the strategies that relatives use to promote their family member’s treatment compliance go beyond the treatment pressures thus far described in the literature.

**Conclusion:**

This qualitative study supports and conceptually expands prior findings that treatment pressures are not only frequently used within mental healthcare services but also by relatives in the home setting. Mental healthcare professionals should acknowledge the difficulties faced and efforts undertaken by relatives in seeking treatment for their family member. At the same time, they should recognize that a service user’s consent to treatment may be affected and limited by strategies to promote treatment compliance employed by relatives.

**Supplementary Information:**

The online version contains supplementary material available at 10.1186/s12888-024-05907-9.

## Background

When caring for a person with a serious mental health condition like schizophrenia or bipolar disorder, relatives and mental healthcare professionals may encounter situations in which they believe that psychiatric treatment is beneficial or necessary, but the person with a serious mental health condition opposes treatment. In these situations, mental healthcare professionals sometimes resort to legally regulated interventions such as involuntary commitment or treatment, which are referred to as formal coercion [1]. Furthermore, mental healthcare professionals frequently exert so-called treatment pressures to promote service users’ compliance^1^ with recommended treatment [3]. Treatment pressures are exerted through various communicative strategies that aim to direct service users’ decision-making, such as persuasion, interpersonal leverage, inducements or threats [4]. These communicative strategies are also discussed as “psychological pressures” and “informal coercion” in the literature [5–7].

Mental healthcare professionals perceive treatment pressures as effective in increasing treatment compliance and avoiding a worsening of symptoms. Furthermore, they report that treatment pressures may prevent formal coercion [8]. At the same time, treatment pressures can also have negative effects, including a potential impairment of the therapeutic relationship [9, 10]. Moreover, treatment pressures may invalidate service users’ consent to treatment by compromising the voluntariness [5], which is a requirement for valid informed consent [11].

Research indicates that not only mental healthcare professionals exert treatment pressures to promote treatment compliance but also relatives and friends. In several qualitative interview studies, service users reported to have experienced persuasion, interpersonal leverage, inducements, and threats from relatives and friends [5, 12–14]. Furthermore, in an interview-based survey with service users, Redlich and Monahan found that relatives and friends are the second most common source of pressure to adhere to treatment within community mental health services after healthcare professionals [15].

While the use of treatment pressures within mental healthcare services is relatively well-documented, research on treatment pressures exerted by relatives is still scarce [16, 17] and no study to date has investigated the perspective of relatives. Furthermore, little is known about what other strategies relatives may use to promote the treatment compliance of their family member with a serious mental health condition (henceforth: family member).

This scarcity of research on treatment pressures and other strategies employed by relatives contrasts with the substantial amount of informal care that many relatives provide for family members [16, 18, 19]. Relatives play an important role in their family member’s pathway to care [20–22] and are often involved in the initiation of involuntary hospital admission [23–26]. The admission-seeking process is experienced as challenging by many relatives [27–30].

The aim of this study was to answer the following research questions: Which types of treatment pressures do relatives use? Which other strategies do relatives use to promote compliance with psychiatric treatment among family members with a serious mental health condition? How do treatment pressures relate to these other strategies?

## Methods

The analysis presented in this article is based on data obtained in a qualitative interview study conducted with relatives of people with a serious mental health condition in Germany. It is reported according to the Standards for Reporting Qualitative Research [31]. The study design followed grounded theory methodology according to Corbin and Strauss [32]. We chose grounded theory methodology for this study because our aim was not to merely examine whether the treatment pressures described by Szmukler and Appelbaum [4] are used by relatives. Rather, we aimed to explore more openly which strategies relatives use to promote their family member’s treatment compliance and how treatment pressures relate to any other strategies.

The researchers in the team have backgrounds in medical ethics, medicine, philosophy, psychiatry, and sociology. The study received ethics approval from the research ethics committee of the medical faculty of Ruhr University Bochum, registration number 18-6584-BR. All participants received written and oral information about the study details and gave written informed consent before participation.

Information on the study was distributed via flyers in a local psychiatric hospital and via email distribution lists of local and national relatives’ self-help groups. Interested participants contacted us on their own initiative via email or telephone. Inclusion criteria were having a family member with a psychiatric diagnosis and the family member having experienced formal coercion. We specified the latter as an inclusion criterion because we were interested in situations in which relatives and their family member have opposing views on the necessity of treatment, and these situations more likely have occurred if experiences with formal coercion (e.g., involuntary commitment or treatment) have been made. Being under 18 years old was an exclusion criterion. We assessed whether potential participants fulfilled the inclusion criteria by means of self-report. In line with grounded theory methodology, we used theoretical sampling, partially supplemented with snowball sampling. Table 1 offers a detailed description of the sample.

**Table 1:** Sample characteristics

Dimension	Description
Relation to the family member*	
Mother	8
Father	2
Husband	2
Brother	1
**Age [years]**	47–68
Gender*	
Female	8
Male	4
Reported diagnosis of the family member*	
Psychotic disorder	8
Bipolar disorder	2
Personality disorder	2
**Length of being a relative of the family member [years]**	2.5–20
Living in the same household at the time of interview*	
Yes	4
No	9
**Contact method**	
Relatives’ self-help group	8
Flyer in local psychiatric hospital	1
Snowball sampling	2

* We conducted a total number of 11 interviews. Aggregate numbers exceed 11 because one interview was a dual interview conducted with the mother and father of a service user and one participant had two family members with a serious mental health condition

Semi-structured interviews were conducted by CH and SP (*n* = 10) or CH and SE (*n* = 1) between October 2019 and January 2020 in Germany. The data reached theoretical saturation after 11 interviews. According to the participant’s preference, interviews took place in their homes, workplaces, or public places, and lasted between 45 and 120 min, with a mean length of 78 min. Based on an interview guide (see Supplementary Material), we asked participants open narrative questions about their relationship with their family member, challenging situations and their way of handling these, their evaluation of mental healthcare services, and suggestions for improvement. Interviews were audio-recorded and transcribed verbatim by an external transcription service and CH. All transcripts were pseudonymized by CH.

The interviews were analyzed by CH and SP using the software MAXQDA 2020. In line with grounded theory methodology [32], our analysis consisted of three steps: (1) open, (2) axial and (3) selective coding. During open coding, we assigned codes describing how relatives promote the treatment compliance of their family member to all passages of the transcripts. These codes were developed inductively but against the background of our previous clinical and theoretical knowledge about treatment pressures. Each transcript was coded by either CH or SP. During axial coding, we added, merged, adjusted, and rearranged codes by comparing them within and across transcripts in an iterative process. As part of this process, we moved from a description of relatives’ dealings to a theorization of underlying strategies to promote treatment compliance. Each researcher focused on the transcripts that had previously been coded by the other researcher and cross-checked their codes for intersubjective comprehensibility. Memos were written to describe the meaning of the codes and to draft working hypotheses. During selective coding, we further theorized the data by identifying conceptual similarities and differences between the various strategies and developed working assumptions to identify core categories of strategies. CH and SP regularly discussed each step of the data analysis and discussed differences in interpretation to improve the analysis. Provisional and final results were discussed with the research team. We did not return transcripts or results to interview participants for member checking.

## Results

Our analysis showed that relatives used various strategies, which included treatment pressures, to promote the treatment compliance of their family member with a serious mental health condition. Relatives commonly employed these strategies when they noticed a change in their family member’s behavior, an intensification of known symptoms or the family member’s discontinuation of medication. Relatives often waited and observed the situation before they interfered, especially at the onset of the mental health condition when relatives were unsure about how to interpret their family member’s behavior.

Relatives emphasized that they tried to initiate treatment or promote compliance out of concern for the family member and, to a lesser extent, other members of the family. Relatives proceeded in steps in their application of the various strategies so as not to lose touch with their family member. Furthermore, regarding initiating the process of hospital admission, relatives often deliberated about how to proceed and which strategies to use because they experienced the process as highly challenging.

When reflecting on situations in which participants initiated involuntary hospital admission or used strategies that they felt involved a lot of pressure, participants often used “one” instead of “I” as the grammatical subject. This may be interpreted as a way of distancing themselves from the action and hence reflects the emotional difficulty and discomfort that relatives experience in these situations.

Based on shared conceptual characteristics, we categorized the strategies we identified in our analysis into three general approaches used by relatives to promote treatment compliance. These approaches are 1) influencing the decision-making of the family member, 2) not leaving the family member with a choice, or 3) changing the social or legal context of the decision-making process. Table 2 gives an overview of the strategies identified within the three approaches. We provide a more detailed description of our analysis below.

**Table 2:** Overview of approaches and corresponding strategies that relatives employ

Strategy		Definition
**Influencing the decision-making of the family member**		
A) Influencing the epistemic basis of the decision		
	Persuasion	Presenting rational arguments to convince the family member to take a certain course of action
	Unwelcome prediction	Pointing out negative consequences that are reasonable to expect given the available evidence
	Nudging	Changing the choice environment to facilitate a certain choice
	Selectively withholding information	Withholding specific information that could lead to the family member taking an undesirable course of action
	Deception	Inducing false beliefs to achieve a certain goal
B) Reducing barriers to compliance		
	Providing support	Giving emotional or practical support to facilitate treatment
	Accommodating preferences	Accommodating the family member’s preferences to make them more comfortable with accepting treatment
C) Influencing the consequences of the decision		
	Interpersonal leverage	Announcing a change in emotional attitude in case the family member does not take the desired course of action
	Inducements	Proposing making the family member better off when they take the desired course of action
	Threats	Proposing making the family member worse off when they do not take the desired course of action
**Not leaving the family member with a choice**		
	Confronting the family member with a decision	Making a decision without the family member’s involvement and informing them of this decision
	Limit setting	Clearly disapproving of or reprimanding certain crisis-related behavior to highlight that treatment is deemed necessary
	Provocation	Deliberatively provoking an escalation of a situation so that legal criteria for involuntary hospital admission are fulfilled
**Changing the social or legal context of the decision-making process**		
	Involving others	Changing the social context by asking mental healthcare professionals or friends to talk to the family member
	Arranging legal guardianship	Changing the legal context by initiating an application for legal guardianship

### Influencing the decision-making of the family member

#### A) Influencing the epistemic basis of the decision

##### Persuasion

Our analysis showed that relatives use persuasion most frequently and as their first option. Relatives described how they, often repeatedly, tried to convince their family member to take a certain course of action by presenting them with arguments speaking in favor of acting in the desired way, as recounted by this mother:

And I said this, for example: “Listen, when you took pills, we could talk to you quite reasonably. Uh, we could also help you. But like this, we can’t.” (Participant 8).

Relatives noted, however, that during a crisis, rational argumentation was often unsuccessful.

##### Unwelcome prediction

Unwelcome predictions involve announcing negative consequences that can reasonably be expected to occur based on the evidence available if the family member does not act in the desired way. Our analysis showed that relatives made unwelcome predictions involving the prospect of formal coercion, such as telling their hospitalized family member that forced medication would soon need to be used if they did not take it voluntarily. As another example, consider the following situation in which the wife of a participant was on a home visit during her hospitalization and refused to return to the hospital:

Of course, I tried to convince my wife and when I was on the phone with the hospital, I explained to her: the hospital says you have to come back and they say, if you don’t go voluntarily, then I should call the police. (Participant 1)

By making unwelcome predictions, relatives merely inform their family member about the expected negative consequences without intentionally bringing these about. For this reason, making unwelcome predictions is a strategy that changes the epistemic basis rather than the actual circumstances of the family member’s decision.

##### Nudging

Participants also used more subtle and indirect ways of influence because explicit attempts to influence decision-making were often fended off by their family member. Changing the choice environment, for example by selectively making specific information available, was used as a nudge to facilitate the desired choice without making one’s goal explicit. One participant described that when he was trying to motivate his family member to seek professional help for her mental health condition, he would intentionally leave books about mental health conditions on the coffee table or tell stories about other people struggling with mental health conditions.

##### Selectively withholding information

Relatives sometimes selectively withheld information from their family member because they were worried that having that information would dissuade the family member from taking the desired course of action. For instance, one participant said he was unsure about whether his wife was aware that she had to take her medication for her mental health condition or if she just took it out of routine. Additionally, he suspected that the occasional stomach problems she reported may be caused by her medication. When asked if he discussed that with her, he answered:

No, especially since I am very happy that she takes the tablets relatively voluntarily, or actually voluntarily, except when she forgets, uh, because, as I said, she suffers very much from, uh, stomach problems. And if she were to get the idea that these might be caused by the medication, then perhaps her compliance with taking the pills would become more difficult again and, therefore, I don’t even want to bring up the discussion. (Participant 1)

##### Deception

In some instances, participants described courses of action that amount to deception. Deception involves intentionally inducing false beliefs in a person to influence her decisions. One participant described that his son constantly locked himself in his room and never came out when anyone was home. One day, a judge needed to see and assess his son for the arrangement of legal guardianship. To make that possible, the family pretended that no one was at home to lead their family member to leave his room. In fact, the father hid in the house and, in this way, once the son had left his room, was able to replace the door handle on his son’s door with one that cannot be locked, thus preventing his son from locking himself in again.

#### B) Reducing barriers to compliance

##### Providing support

Participants further reported offering their support to reduce potential barriers to utilizing treatment. They would, for example, offer to go to the hospital together during a crisis or regularly accompany their family member to scheduled appointments. Driving family members to treatment appointments so that they do not have to rely on public transport was another way of providing support, as illustrated by this mother:

I’m not going to say, “You go to some common or garden psychologist because I don’t have time to drive.” […] We sometimes drove twice a week. (Participant 3)

##### Accommodating the family member’s preferences

Another way of reducing barriers to compliance was trying to accommodate the family member’s preferences to make the family member more comfortable with accepting treatment. One participant recounted that, during one crisis, his wife was hostile towards men. Before going to the hospital, he called to see who was on duty in the emergency room:

I had made sure that it was a woman […] and then we went there […] only then went there together. (Participant 4)

#### C) Influencing the consequences of the decision

##### Interpersonal leverage

Our analysis showed that another way for relatives to influence the decision-making of their family member was by exerting interpersonal leverage. The latter builds on the emotional bond between relatives and their family members and amounts to announcing a change in one’s emotional attitude if the family member does not take the desired course of action. Relatives, for example, told their family member how worried they are about the family member’s current situation and implicitly or explicitly tied this to their own wish that the family member consult a psychiatrist or take their medication. This is evident in a mother’s description of the interaction with her daughter after her boyfriend’s father had informed the mother about her daughter’s self-harm intentions:

And we talked to her and just said that we got this call and that we were very worried […] And then she said she was going to the hospital herself. (Participant 5)

Expressing significant worry influences the consequences of the family member’s decision in that it holds the implicit implication that the family member would emotionally burden their relative by refusing treatment. In other cases, relatives explicitly announced a change in their emotional attitude, such as getting angry or desiring to no longer be in contact if their family member were to refuse to take the desired course of action. This is illustrated by this brother’s description of an interaction with his sister:

Then we also became angry and wrote: If you don’t stop [your mental health condition-related behavior] now, we don’t want to have anything more to do with you. Then we break off contact. (Participant 10)

##### Inducements

Relatives further used inducements by offering their family member something that makes them better off if they act in the desired way. In the following case, a young man currently had no place to sleep during a psychotic episode. His mother recounted:

She [grandma] then called me and said, “Okay, I took him in tonight but told him only for tonight and tomorrow we’ll go to the hospital together and you’ll let yourself be admitted.” (Participant 6)

Relatives also described making agreements with their family member based on a reciprocal commitment. For instance, when asked by their family member to take them home from the hospital or assisted living facility, the relative agreed to do so only under certain conditions, such as the family member taking medication or finding an alternative living arrangement, as illustrated by the following interview excerpt:

Well, I just made an agreement with her […]. Yes, when I bring her home, that we look for a place in an assisted living facility together and that she goes there when we find one. (Participant 7)

##### Threats

Relatives sometimes used threats by indicating that they will make their family member worse off if the latter does not take the desired course of action. In our analysis, this often involved the prospect of having to move out of the house. One participant, for example, considered telling his wife:

“As long as you act crazy, I will kick you out” (Participant 4).

### Not leaving the family member with a choice

#### Confronting the family member with a decision

Sometimes, relatives simply told their family member what to do or made decisions without their involvement, thus confronting their family member with a decision that had already been made. While this may be frequent in interactions between parents and their children, relatives described this pattern as stretching out to an age where children usually make their own decisions. A mother who registered her reluctant son for an assisted living facility, said:

I just signed him up thinking, “Well, let’s see.” And I informed him of that. (Participant 2)

#### Limit setting

Participants shared that interacting with their family member during a crisis could be exhausting and that they therefore set limits to make their own needs visible. They described clearly expressing disapproval of specific behaviors, which they attributed to a mental health crisis, to their family member and occasionally reprimanding them for such behaviors. One mother recounted how her son, who had his own apartment and was experiencing a psychotic episode, became very aggressive and insulting towards his sister during a family dinner. She recounted:

We then said: “That’s not how we behave here. You cannot act like this. You have to leave.” (Participant 9)

Relatives typically engaged in limit setting to underline their belief that their family member must take the prescribed psychiatric medication or comply with treatment.

#### Provocation

One participant shared how she, out of great worry, desperation and under great moral distress, provoked a situation in which her son appeared to be a danger to her to fulfil the legal criteria for involuntary hospital admission after previous attempts to talk him into seeking treatment had failed. When her son came home one evening after wandering around for days, she locked the door of the house, which he noticed when he wanted to buy cigarettes the next morning. She told him she would accompany him and stalled him when he tried to unlock the door, which she expected would make him aggressive. Upon escalation, she called the police.

### Changing the social or legal context of the decision-making process

#### Involving others

Our analysis revealed that relatives frequently involved others in their attempts to promote their family member’s treatment compliance, which we conceptualized as a strategy to change the social context of the decision-making process. Relatives described asking mental healthcare professionals to talk to their family member. Further, participants called the police or the emergency services in crisis situations, often trying to initiate involuntary hospital admission.

And then the delusion gets more and more extreme and at some point, uh, one calls an ambulance. (Participant 11)

Additionally, participants asked friends of their family member, some of whom were service users themselves, to talk to the family member. This way, participants hoped to get in contact with their family member again through a person of trust or with similar experiences.

#### Arranging legal guardianship

A final strategy that our analysis revealed was relatives initiating an application for legal guardianship of their family member, often to facilitate psychiatric treatment. Legal guardianship changes the legal context and power dynamics of the decision-making process considerably in that involuntary hospital admission and treatment can be arranged more easily. While some participants applied to act as the legal guardian themselves, others preferred an external person to act as the legal guardian because it enabled them to delegate difficult tasks and focus on their role of being a relative. In cases where a third person rather than the relative acts as the legal guardian, this changes not only the legal but also the social context of the decision-making process.

### Theoretical integration of our results

As part of our analysis, we theorized the different ways relatives promote their family member’s treatment compliance into more abstract strategies. Doing so allowed us to identify conceptual similarities and differences between the various strategies. Based on these, we categorized the strategies into three general approaches that relatives use to promote their family member’s treatment compliance: 1) influencing the decision-making of the family member, 2) not leaving the family member with a choice, and 3) changing the social or legal context of the decision-making process.

Our categorization of the various strategies into three general approaches highlights that relatives find different leverage points to promote treatment compliance. The first approach aims to obtain the family member’s consent for treatment through a process of negotiation. By contrast, the second approach aims to avoid negotiation with the family member and denies them the opportunity to give consent. Finally, the third approach aims to promote treatment compliance by influencing the background conditions of the decision-making process rather than the decision-making process itself.

The first approach allows for a further conceptual subdivision. Relatives may influence their family member’s decision-making either by influencing the beliefs they hold or by influencing what is the case. By influencing the family member’s beliefs, relatives influence the epistemic basis of the family member’s decision-making without changing any facts about the world. Relatives may do so by adding or stressing specific information, either verbally (e.g., by using persuasion or uttering an unwelcome prediction) or non-verbally (e.g., by means of nudging), or by selectively withholding information. They may also purposely induce false beliefs to promote treatment compliance (i.e., deception).

In contrast to influencing the beliefs based on which the family member makes their decision, relatives may also change the facts that guide the family member’s decision-making. Relatives may do so by altering the consequences of a certain decision to make that decision more or less attractive to their family member (e.g., by means of interpersonal leverage, inducements, or threats). Alternatively, relatives may reduce the barriers to making a certain decision and thus influence the preconditions of that decision (e.g., by providing support or by accommodating preferences).

Overall, our results highlight that the strategies that relatives use to promote their family member’s treatment compliance go beyond the treatment pressures thus far discussed in the literature. The aim of treatment pressures is to influence someone’s decision-making. In our analysis, we found that relatives influence their family member’s decision-making not only by means of persuasion, interpersonal leverage, inducements, or threats, but also by other strategies included in approach one. Furthermore, our development of the three different approaches demonstrates that influencing the family member’s decision-making is not the only leverage point for relatives to promote treatment compliance.

## Discussion

This qualitative study with relatives of people with a serious mental health condition investigated the strategies that relatives use to promote their family member’s compliance with psychiatric treatment. We categorized the different strategies developed in our analysis into three approaches: 1) influencing the decision-making of the family member, which includes treatment pressures previously described in the literature, 2) not leaving the family member with a choice, and 3) changing the social or legal context of the decision-making process.

Our study confirms findings from previous studies conducted with service users and mental healthcare professionals that relatives frequently exert treatment pressures [5, 12–15]. Szmukler and Appelbaum’s [4] taxonomy of treatment pressures focuses on strategies influencing the decision-making of service users and was developed in the context of mental healthcare services. It includes persuasion, interpersonal leverage, inducements, and threats, which we also found in our analysis.

Yet, our analysis highlighted that the distinction between inducements and threats is less clear when exerted by relatives as opposed to mental healthcare professionals. Building on Wertheimer [33, 34], Szmukler and Appelbaum [4] distinguish between inducements and threats with reference to a moral baseline that is determined by the moral rights of the proposal’s recipient and describes the way the recipient ought to be treated by the proposer. Threats involve indicating that one will make the recipient worse off than they morally ought to be if they do not take the desired course of action, whereas inducements involve indicating that one will make the person better off, or at least no worse off, than they morally ought to be if they take the desired course of action. The distinction between inducements and threats is crucial because, in the philosophical debate, threats are commonly considered coercive while inducements are not [4, 33, 34].

Dunn et al. [35] suggest using mental healthcare professionals’ duties of care as the moral baseline to distinguish between inducements and threats in the context of mental healthcare. This determination of the moral baseline, however, does not provide guidance in evaluating treatment pressures exerted by relatives, because the scope of relatives’ duties of care is typically rather vague. During our analysis, for example, the following questions arose: Does an adult family member have a moral right to live with their relatives? Is it an inducement or a threat if relatives propose that their family member can only live with them if they comply with treatment? Do relatives have a moral duty to keep in touch with their family member under all circumstances?

Our explorative research design allowed us to further develop strategies to influence the family member’s decision-making not included in Szmukler and Appelbaum’s [4] taxonomy of treatment pressures. *Unwelcome predictions* are defined as “acts that resemble ‘coercive’ threats” by Szmukler and Appelbaum [4, p. 237] and are also discussed as *warnings* within psychiatric ethics [36]. *Withholding information* has been critically discussed within medical ethics as informational manipulation that may compromise informed consent [11]. Seale et al. [37] have pointed out that psychiatrists sometimes withhold information or even lie to service users because they fear honesty about adverse effects will hinder medication compliance. This relates to our findings regarding relatives selectively withholding information.

Going beyond prevailing research on treatment pressures, we also generated positive strategies to promote treatment compliance by influencing the family member’s decision-making, such as relatives’ efforts to reduce barriers to treatment compliance through *providing support* and *accommodating preferences.* Whenever feasible, these positive strategies should be preferred over other strategies as they do not constrain the voluntariness of consent.

We further identified not leaving the family member with a choice as an approach used by relatives. Similar to our strategy *confronting the family member with a decision*, Klingemann et al. [14] used the label *someone else’s decision* to describe service users’ experience of decisional power being taken away from them by mental healthcare professionals or family members. While Pelto-Piri et al. [13] discussed mental healthcare professionals *using a disciplinary style* as a way to enhance compliance with social rules, we found relatives use *limit setting* additionally to signal that they deem treatment to be necessary.

Our finding that relatives sometimes change the social or legal context of the decision-making process resonates with qualitative studies with service users which highlight how the relationship between the interacting parties and possible power imbalances impact decision-making and the perceived coerciveness of communicative interactions [5, 38]. Additionally, Andersson et al. [39] describe that psychiatric nurses also use the strategy of *involving others*.

The different strategies relatives use to promote treatment compliance of their family member must be considered against the background of the organization of mental healthcare services and legal regulation of coercive interventions. For example, German mental healthcare services are, to a large extent, offered in inpatient settings, while other countries offer more outpatient and community services [40]. Furthermore, the legal criteria for involuntary commitment and treatment vary across jurisdictions [41]. While a range of jurisdictions allow for so-called community treatment orders [42], these are not used in Germany [43, 44]. Community treatment orders tie treatment in the community to the condition of treatment compliance and facilitate the process of hospital admission when necessary [45]. Accordingly, the existence of community treatment orders will likely influence the strategies relatives use to promote treatment compliance.

Furthermore, it is important to understand why relatives use the described strategies to promote their family member’s treatment compliance. We provide a detailed analysis of this in another forthcoming paper, which highlights that relatives employ strategies to promote treatment compliance as part of assuming responsibility for their family member. Relatives assume this responsibility due to emotional and familial bonds with their family member, societal expectations, and being transferred responsibility from the mental healthcare system. In line with our findings, other qualitative studies with relatives of people with a serious mental health condition have stressed the incisive role of emotional and familial bonds with their family member in relatives’ effort to promote treatment compliance [46, 47]. Furthermore, previous research has highlighted that responsibility is often placed on relatives by the mental healthcare system, especially with an increasing deinstitutionalization of mental healthcare [17, 19]. For example, in a qualitative interview study relatives described they felt responsible for providing care and promoting treatment compliance because mental health services did not take over these tasks [48].

### Strengths and limitations

By investigating the perspective of relatives of people with a serious mental health condition, this study adds an important perspective that has so far been missing in research on treatment pressures. Another significant strength of our study is that our explorative research design allowed us to develop strategies that go beyond Szmukler and Appelbaum’s [4] influential taxonomy of treatment pressures.

A possible limitation of our study concerns the transferability of our results to contexts with significantly different organization of the mental healthcare services or mental health laws. As discussed above, these contextual factors may influence the strategies used. Accordingly, additional research in countries with differently structured mental healthcare services and mental health laws is needed. Another possible limitation relates to the large proportion of mothers in our sample. This, however, mirrors the reality of the unequal distribution of informal care work.

## Conclusion and implications

This qualitative study supports prior findings that not only mental healthcare professionals but also relatives of people with a serious mental health condition exert treatment pressures. It further highlights that relatives use a variety of strategies to promote their family member’s compliance with psychiatric treatment that go beyond treatment pressures previously described. Mental healthcare professionals should acknowledge the difficulties faced and efforts undertaken by relatives in seeking psychiatric treatment for their family member and, accordingly, take their concerns seriously. Simultaneously, professionals should recognize that a service user’s consent to treatment may be compromised by strategies to promote treatment compliance employed by relatives. The taxonomy of strategies identified in our study can provide guidance to professionals in assessing whether this is the case.

### Electronic supplementary material

Below is the link to the electronic supplementary material.

Supplementary Material 1

## Data Availability

We cannot share research data publicly as individual privacy could be compromised. Research data are available from the corresponding author upon reasonable request.

## Footnotes

1 We recognize that, instead of “compliance”, alternative terms such as “adherence” or “concordance” should generally be preferred to emphasize the importance of actively involving service users in the decision-making process. However, in the context of treatment pressures, we found it more appropriate to use “compliance”, which describes the extent to which a service user follows recommended treatment [2].
