# Supplementary material for: Strategies to promote treatment compliance: a grounded theory study with relatives of people with a serious mental health condition
Source: BMC Psychiatry. 2024 Jul 8;24:490. doi: 10.1186/s12888-024-05907-9 (PMC11229214; doi:10.1186/s12888-024-05907-9)
Supplement: Supplementary file 1 — Supplementary Material 1 [file 12888_2024_5907_MOESM1_ESM.docx]

# Supplementary Material

## Interview Guide

1. Introduction

- Thank the participant for their willingness to participate, explain the recording device and switch it on
- Introduction of the interviewers
- Explanation of the procedure, reference to confidential treatment of the interview material/pseudonymization, explanation of the topic
- Note on time: interview takes approx. 60 – 90 minutes
- Answer any organizational questions that may arise

1. Main part

| **Leading question** (narrative prompt) | **Check: Was this mentioned?** (memo for possible follow-up questions) | **Specific questions** (to be invoked at an appropriate time) | **Questions to steer and maintain the conversation** |
| --- | --- | --- | --- |
| **Part I:** **Narrative questions prompting memory:**  **1.** How did it come about that you sought out the XY self-help group for relatives of people with a serious mental health condition?  **2.** What is your relationship with your family member like?  **3.** Were there any situations that you found particularly challenging? [If yes] What kind of situations were these? Tell us about one such situation - what happened there?  **4.** Do you remember a situation that was just as challenging but which turned out differently? [If yes] Tell us about the situation - what happened? | Which people belong to your family and social network?    Quality/intensity of relationships    Is the social environment seen as supportive?    Relevant changes before and after the diagnosis?    Challenging situations = process or sudden/unexpected event?    What role do treatment pressures play in the interaction with hospital staff (bidirectional)?    Is the relationship with the family member associated with coercion or treatment pressures? | What was your relationship like before and after your family member's diagnosis?    Which people were involved in situation XY?    How did situation XY come about? What happened before? What happened afterwards?    How did you feel in the situation? | Can you say anything more about this?    And then?    What happened next?    How did you feel then?    Nonverbal communication |
| **Part II: Reflecting and summarizing questions:**    **5.** Looking back, how would you now deal with the challenging situations you described earlier?    **6.** How do you feel about the decisions that your family member has made so far in dealing with his/her serious mental health condition (e.g., taking or not taking medication; seeking or refusing professional support)? | Assigning blame? For what? If so, to whom? Participant, their family member, the mental health condition, others?    What feelings are associated with certain experiences?    Does the participant ascribe agency to his/herself or expresses a feeling of helplessness/powerlessness?    What is the participant's perception of a serious mental health condition?    Does the participant feel exposed to the family member or their mental health condition?    Is there awareness of the treatment pressures exerted or not?    Are treatment pressures (which forms specifically) accepted or approved of?    Are treatment pressures (which forms specifically) rejected?  What are alternatives to using treatment pressures? | In your opinion, can treatment pressures be positive/negative? If so, in what way?    In what contexts and situations do you consider the use of treatment pressures to be positive/negative (e.g., danger to self or others)? |  |
| **Part III: Attitude and evaluation questions**    **7** How would you rate the existing support services for people with a serious mental health condition and their relatives?    **8.** Do you have any suggestions for further support or other support services?    **9.** How do you rate the cooperation with the hospital staff? What suggestions do you have for improvement? | Are existing support offers assessed as helpful, useless, insufficient, etc.?    Is there room for improvement to reduce the use of (in)formal coercion? |  |  |
| **Part IV: Conclusion**    9. Have we forgotten anything that you would like to mention?    10. How did you find the interview? |  |  |  |

1. Conclusion

- Record demographic information using the accompanying questionnaire
- Thank the participant for their participation and make reference to contact details for possible follow-up questions he/she might have
